# Effects of Trunk Extension-Based Inspiratory Muscle Strengthening on Respiratory Function, Balance, and Gait in Patients with Stroke: A Randomized Controlled Trial

**DOI:** 10.3390/jcm15052017

**Published:** 2026-03-06

**Authors:** Kwang-Bin An, Hye-Joo Jeon, Yu-Sik Choi, Soo-Yong Lee, Woo-Nam Chang

**Affiliations:** 1 Department of Physical Therapy, Bobath Hospital, Hanam 12927, Republic of Korea; mrbin9827@naver.com; 2 Department of Physical Therapy, U1 University, Yeongdong 29131, Republic of Korea; bluestar@u1.ac.kr; 3 Department of Physical Therapy, College of AI-Bio Convergence, Yongin University, Yongin 17092, Republic of Korea; cus6208@naver.com; 4 Department of Physical Therapy, Severance Rehabilitation Hospital, Yonsei University, Seodaemun-gu, Seoul 03722, Republic of Korea; ywlee2000213017@hanmail.net

**Keywords:** balance, gait, inspiratory muscle strengthening, respiratory function, stroke

## Abstract

**Objectives:** This study investigated the effects of trunk extension-based inspiratory muscle strengthening on respiratory function, balance, and gait in patients with stroke. **Methods:** Thirty stroke patients were randomly assigned to the study group (*n* = 15) or control group (*n* = 15). The study group performed inspiratory muscle strengthening exercises in a trunk extension posture, while the control group received conventional inspiratory muscle training. Both groups trained five times per week for six weeks. Outcome measures included maximal inspiratory pressure (MIP), maximal inspiratory flow rate (MIFR), maximal inspiratory volume (MIV), peak expiratory flow (PEF), forced expiratory volume in 1 s (FEV_1_), Berg Balance Scale (BBS), weight distribution ratio (WDR), limits of stability (LOSs), Timed Up and Go (TUG), gait velocity, cadence, and stride length. **Results:** The study group showed significantly greater improvements in respiratory parameters (MIP, MIFR, MIV, PEF, FEV_1_) and functional outcomes (WDR, LOS, BBS, TUG, gait velocity, cadence, stride length) compared to the control group. **Conclusions:** Trunk extension-based inspiratory muscle strengthening effectively improves respiratory function, balance, and gait in stroke patients, and may serve as a valuable addition to stroke rehabilitation programs.

## 1. Introduction

Problems with respiratory muscle control in patients with stroke can lead to insufficient contractile strength and decreased rhythmic movement patterns and lung capacity [1]. Prolonged paralysis of the respiratory muscles, including the diaphragm, prevents adequate inflation of the lungs and thoracic cage, leading to shortening and fibrosis of the tissue surrounding the thoracic cage and decreased ventilatory capacity [2]. Furthermore, postural stability is affected, leading to difficulty in postural control and an increased risk of falls [3,4,5,6]. In particular, long-term bedridden individuals or wheelchair users develop trunk muscle weakness and imbalances [7] and motor activities that rely on compensatory postural strategies [8]. Compensatory postural strategies in the trunk increase asymmetric flexion of the trunk and coordination of the paretic arm, which is unlikely to provide sufficient stability for functional movements [9]. Difficulties with trunk extension and symmetry make normal activation of the respiratory muscles difficult and are associated with weakening of the pectoralis major and respiratory muscles [1,10].

Inspiratory muscle training is an effective and safe intervention for enhancing respiratory function, improving quality of life, reducing dyspnea, and improving postural stability and balance in patients with stroke [11,12,13]. Improving the function of the respiratory muscles requires an increase in muscle strength, which requires respiratory muscle strengthening [12]. Strengthening respiratory muscles reduces respiratory muscle fatigue and improves exercise performance [14]. The beneficial effects of inspiratory muscle training may be explained by stroke-related alterations in respiratory mechanics. In patients with stroke, asymmetrical trunk posture and respiratory muscle weakness may mechanically restrict thoracic expansion and reduce ventilatory efficiency [1,9]. Impaired coordination and decreased strength of the respiratory muscles contribute to reduced lung volumes and functional capacity [1,10]. Targeted inspiratory muscle strengthening may enhance diaphragmatic contractility, improve chest wall mobility, and promote more symmetrical activation patterns, thereby contributing to improvements in pulmonary function and functional performance [9,10]. Petrovic et al. [15] found that inspiratory muscle training improves ventilatory capacity and respiratory muscle function. Furthermore, Illi et al. [16] found that inspiratory muscle training improves lung function. The diaphragm is the most important respiratory muscle and is attached to the ribs and lumbar vertebrae, helping with postural stability and control [17,18]. Further activation of the diaphragm through inspiratory muscle training can improve postural control and functional movement [19].

Routine physical therapy for patients with stroke that focuses solely on physical recovery is insufficient for improving respiratory function [20]. Some studies have suggested that physical therapy that includes interventions for improving respiratory function is more effective in improving the function of patients with stroke [21]. Furthermore, respiration has been reported to be correlated with gait in patients with stroke, with decreased respiratory function adversely affecting gait [22]. Several studies have assessed the efficacy of inspiratory muscle training for improving balance and respiratory function recovery in patients with stroke [10,12,23,24]. However, research on inspiratory muscle training that considers functional postures optimized for respiratory muscle activity, including the diaphragm, remains limited.

Therefore, this study investigated the effects of inspiratory muscle strengthening based on trunk extension posture on respiratory function, balance, and gait in patients with stroke. Specifically, this study compared trunk extension-based inspiratory muscle training with conventional training to determine whether optimizing trunk posture during inspiratory muscle strengthening would result in greater improvements in respiratory parameters, postural stability, and gait performance. We hypothesized that inspiratory muscle training performed in a trunk extension posture would lead to superior functional outcomes compared with conventional intervention.

## 2. Materials and Methods

### 2.1. Participants

This study was approved by the Institutional Review Board of Yongin University (approval No. 2204-HSR-254-3), and written informed consent was obtained from all participants. The trial was registered with the Clinical Research Information Service (CRIS; Registration No. KCT0009791). The study included 30 patients with stroke who were hospitalized at B Hospital, Seongnam-si, Gyeonggi-do, Korea, between July 2022 and June 2023. The patients voluntarily agreed to participate in the study after being fully informed of its outline and purpose. The sample size was calculated using G*Power (version 3.1.9.2; Heinrich-Heine-Universität Düsseldorf, Düsseldorf, Germany) [25] based on an effect size derived from a previous pilot study conducted by the authors. The calculation was performed using an independent samples *t*-test for between-group differences in change scores, with a significance level of 0.05, statistical power of 0.95, and an effect size of 1.61. The minimum required sample size was 20 participants (10 per group). Considering a potential dropout rate, 30 participants were recruited. The inclusion criteria were as follows: (1) patients diagnosed with stroke by a physician; (2) those aged between 15 and 85 years; (3) those who were able to walk independently for at least 30 m with or without an assistive device; (4) those with no history of severe cardiovascular or respiratory disease; (5) those who scored ≥24 on the Korean version of the Mini-Mental State Examination (MMSE-K). All participants or their legal guardians provided written informed consent prior to participation in accordance with Institutional Review Board approval.

Participants were excluded if they: (1) had other neurological disorders in addition to stroke; (2) had severe musculoskeletal impairments affecting trunk posture or gait performance; (3) had unstable medical conditions that could interfere with participation in the training program; (4) were currently participating in other respiratory rehabilitation or experimental interventions.

**Figure 1:**
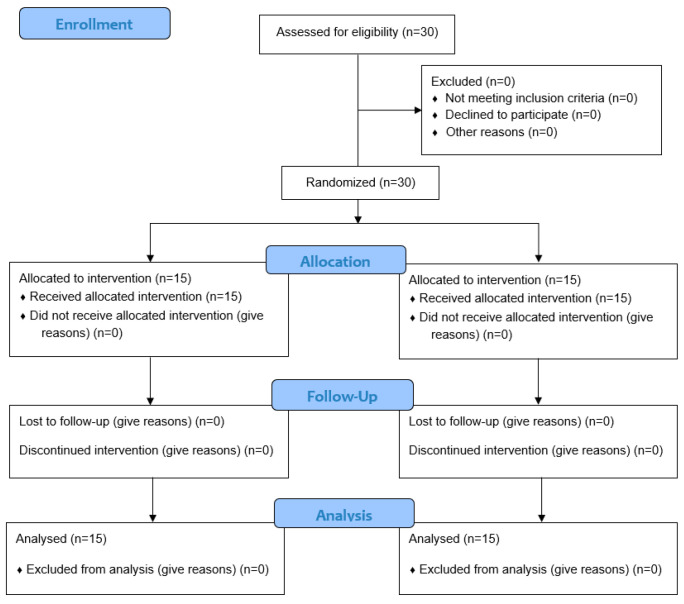
Diagram of the study process.

Participants were allocated using stratified randomization based on sex (16 males, 14 females) and side of hemiplegia (right, *n* = 14; left, *n* = 16), in accordance with recommended procedures for randomized clinical trials [26,27]. Within each stratum, participants were randomly assigned to either the study group (Group A) or the control group (Group B) using sealed opaque envelopes containing allocation cards. Equal numbers of A and B cards were prepared within each stratum, thoroughly mixed, and drawn individually by participants to ensure balanced group allocation. As a result, 15 participants were assigned to the study group and 15 to the control group, with equal distribution according to sex and side of hemiplegia (Figure 1).

The intervention period lasted six weeks (five sessions per week, 30 min per session). Outcome assessments were performed before and after the six-week intervention period. To enhance objectivity, outcome assessors were blinded to group allocation.

### 2.2. Intervention

The interventions in the study group were adapted and modified from those proposed by Sutbeyaz et al. [12] and An et al. [24]. Participants began treatment in a seated position with lumbar and pelvic support on a height-adjustable treatment table. A treatment table was placed in front of them to support the upper extremities, and they were instructed to maintain an upright trunk extension posture throughout the training. Verbal cues were provided by the therapist to facilitate proper posture and thoracic expansion.

Inspiratory muscle training was performed using a POWERbreathe K5 (POWERbreathe International Ltd., Warwickshire, UK). The total training time was 30 min per session. Each session consisted of two 15 min training blocks separated by a 5 min rest period. Participants wore nasal clips, and a personalized mouthpiece was fitted tightly to prevent air leakage. They were instructed to exhale maximally and then inhale quickly and forcefully against the set resistance to ensure effective diaphragmatic activation. The initial resistance was set at 40% (auto mode–very light) of the participant’s maximal inspiratory pressure (MIP) and was gradually increased up to 60% (auto mode–moderate) according to individual tolerance.

**Figure 2:**
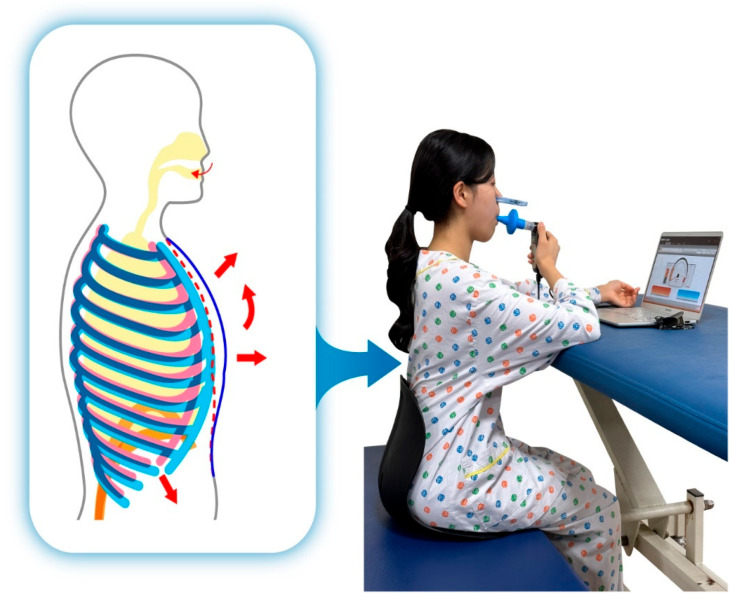
Inspiratory muscle training.

The intervention was performed for 30 min per day, five times per week, for 6 weeks under therapist supervision. The training procedure was demonstrated prior to the intervention to ensure familiarity. If participants experienced dizziness or excessive respiratory discomfort, the session was immediately paused until stabilization (Figure 2).

The control group performed general trunk control training consisting of bridge exercises, sit-ups, trunk flexion and extension, trunk rotation, and upper extremity extension exercises. Each session included 5 min of warm-up, 20 min of the main exercises, and 5 min of cool-down, totaling 30 min per day, five times per week for 6 weeks. All exercises were supervised by a therapist to ensure correct performance and safety.

### 2.3. Measurement

Pulmonary function was assessed by measuring both inspiratory and expiratory performance. For the inspiratory function test, maximum inspiratory pressure (MIP), maximum inspiratory flow rate (MIFR), and maximum inspiratory volume (MIV) were measured using a POWERbreathe K5 device (International Ltd., Warwickshire, UK). Each subject was seated comfortably and used a personalized mouthpiece during the test. Prior to measurement, the procedure was explained and demonstrated to ensure proper understanding and execution. The device demonstrated excellent reliability, with intrarater intraclass correlation coefficients (ICCs) ranging from 0.959 to 0.986, and interrater ICCs ranging from 0.933 to 0.985 [28].

For the expiratory function test, spirometry was conducted using a microlife PF-200 spirometer (Microlife Corp., Widnau, Switzerland) to evaluate peak expiratory flow (PEF) and forced expiratory volume in one second (FEV1). Subjects were instructed to sit comfortably, inhale fully, and then exhale as forcefully as possible into the device. Each participant completed three trials, and the average value was used for analysis.

To evaluate balance, both the weight distribution ratios (WDRs) and limits of stability (LOSs) were assessed using the Biorescue platform (BRP, Rm Ingenierie, Marseille, France) in a standing position. The WDR was calculated by having subjects stand barefoot on the platform for one minute, with their gaze fixed straight ahead. The absolute value of the difference in load between the left and right sides was used as the WDR. For the LOS measurement, participants were instructed to voluntarily shift their center of gravity in accordance with on-screen directional prompts. Trials were repeated if either foot lost contact with the platform. Each measurement was performed three times, with a 3 min rest interval between trials, and the mean value was recorded.

In addition, balance was also evaluated using the Berg Balance Scale (BBS), which consists of 14 items scored from 0 to 4, with a maximum score of 56. This scale has demonstrated excellent reliability in stroke patients, with reported intrarater and interrater correlation coefficients of r = 0.98 and r = 0.99, respectively [29].

To assess gait performance, the Timed Up and Go (TUG) test and the 10 m walk test were conducted. The TUG test, which has high intrarater (r = 0.99) and interrater (r = 0.98) reliability, was used to evaluate balance, gait speed, and functional mobility [30]. Participants completed the test three times, and the average duration was used for analysis.

The 10 m walk test was performed along a 14 m path, with the first and last 2 m allocated for acceleration and deceleration. Walking time was recorded for the middle 10 m only. This test also demonstrated high reliability (r = 0.95–0.96) [31,32], and the average of three trials was used for subsequent analysis.

### 2.4. Statistical Analysis

All statistical analyses were performed using the Statistical Package for the Social Sciences (SPSS; version 18.0; IBM Corp., Armonk, NY, USA). The Shapiro–Wilk test was used to assess the normality of all variables, and the assumption of normal distribution was satisfied (*p* > 0.05).

Baseline homogeneity between groups was examined using independent samples *t*-tests for continuous variables and chi-square tests for categorical variables. Within-group differences between pre- and post-intervention were analyzed using paired samples *t*-tests, and between-group differences in change values were analyzed using independent samples *t*-tests. All measurement results are presented as means ± standard deviations, and the statistical significance level (α) was set at 0.05.

## 3. Results

### 3.1. General Characteristics of the Patients

No statistically significant differences in general characteristics were observed between the study and control groups (Table 1).

The pre-assessment homogeneity test of the dependent variables for the study and control groups exhibited no statistical difference between the two groups (Table 2).

### 3.2. Comparison of Respiratory Function

In the study group, the maximum inspiratory pressure (*p* < 0.01), maximum inspiratory flow rate (*p* < 0.01), and maximum inspiratory volume (*p* < 0.05) significantly increased after the intervention. The between-group comparison of the study and control groups revealed statistically significant differences in maximum inspiratory pressure (*p* < 0.01), maximum inspiratory flow rate (*p* < 0.01), and maximum inspiratory volume (*p* < 0.05) (Table 3).

Furthermore, in the study group, peak expiratory flow (*p* < 0.05) and forced expiratory volume in 1 s (*p* < 0.05) significantly increased after the intervention. The between-group comparison between the study and control groups revealed statistically significant differences in peak expiratory flow and forced expiratory volume in 1 s (*p* < 0.05) (Table 3).

### 3.3. Comparison of the Balance Ability

In the study group, the weight distribution ratio decreased significantly after the intervention (*p* < 0.01). The between-group comparison of the study and control groups revealed a statistically significant difference in the weight distribution ratio (*p* < 0.01) (Table 4).

Moreover, the limits of stability increased significantly in the study group after the intervention (*p* < 0.01). The between-group comparison of the study and control groups revealed a statistically significant difference in the limits of stability (*p* < 0.05) (Table 4).

In the study group, Berg Balance Scale scores increased significantly after the intervention compared with those before the intervention (*p* < 0.001). The between-group comparison between the study and control groups revealed a statistically significant difference (*p* < 0.05) (Table 4).

### 3.4. Comparison of the Gait Ability

Following the intervention, the study group demonstrated a significant reduction in Timed Up and Go Test scores (*p* < 0.05), whereas no significant between-group difference was observed (*p* > 0.05) (Table 5).

During the 10 m walk test, the study group showed significant improvements in all gait parameters, including gait speed (*p* < 0.05), cadence (*p* < 0.05), and stride length (*p* < 0.01). However, no statistically significant differences between the study and control groups were identified for any of these variables (*p* > 0.05) (Table 5).

## 4. Discussion

After stroke onset, respiratory muscle contraction is approximately 80% of that of normal individuals and is accompanied by muscle weakness and muscle tone abnormalities [33]. Muscle weakness and muscle tone abnormalities in patients with stroke lead to trunk asymmetry and abnormal postural responses, which decrease numerical lung capacity [34]. To address these issues, thorax expansion and ventilation and adequate lung capacity and volume must be maintained, and strength, range of motion, and flexibility of major and accessory inspiratory muscles of the thorax and trunk are required [35].

Inspiratory muscle training is a well-established method for strengthening respiratory muscles [36] and has been shown to improve pulmonary function and gait ability in patients with stroke [37]. Several previous studies have reported improvements in respiratory parameters following inspiratory muscle training. Sutbeyaz et al. [12] demonstrated that 6 weeks of training significantly improved inspiratory muscle function, peak expiratory flow, and forced expiratory volume in 1 s in patients with stroke. Similarly, Messaggi-Sartor et al. [38] reported increased maximal inspiratory pressure after 3 weeks of resistance training at 30% of maximal inspiratory pressure. An et al. [24] observed significant improvements in maximal inspiratory pressure, maximal inspiratory flow rate, maximal inspiratory volume, peak expiratory flow, and forced expiratory volume in 1 s compared with those in a control group. In addition, Kim et al. [39] and Yoo et al. [40] reported significant improvements in peak expiratory flow and forced expiratory volume in 1 s following respiratory muscle training. The improvements in respiratory function observed in the present study are consistent with these previous findings. However, unlike earlier studies that primarily focused on inspiratory muscle loading alone, the current study incorporated trunk extension posture during training. This posture may have facilitated greater thoracic expansion and more effective diaphragmatic activation, thereby contributing to the significant improvements in respiratory function observed in the study group.

Patients with stroke experience difficulty in controlling trunk balance in addition to decreased respiratory function due to weakness in the inspiratory and expiratory muscles, altered muscle tone, and decreased thorax expansion [34]. Alfieri et al. [41] found that patients with stroke showed changes in postural control and decreased postural stability in response to swaying compared with healthy individuals. Jung et al. [42] reported that inspiratory muscle training improved respiration and trunk control in patients with stroke. An et al. [24] found that respiratory muscle training improved balance in a study involving patients with stroke. They reported a statistically significant decrease in the weight distribution ratio and an increase in the limits of stability in the study group after the intervention and statistically significant results in the weight distribution ratio and limits of stability compared with those in the control group. Furthermore, Aydoğan Arslan et al. [23] found that inspiratory muscle training improved respiratory function, trunk control, and balance in patients with stroke. de Almeida et al. [43] reported that training of the inspiratory muscles enhanced inspiratory muscle strength and increased muscle activity of the diaphragm. The diaphragm and inspiratory muscles are closely related to postural control [44,45]. We believe that inspiratory muscle training based on the trunk extension posture improves the strength of the main inspiratory muscles, diaphragm, outer intercostal muscles, auxiliary inspiratory muscles, thoracic ribs, and thyroid, contributing to the postural stability of patients with stroke and thus improving their static and dynamic balance.

The gait speed of patients with stroke depends on the function of the legs and trunk [46,47]. Verheyden et al. [48] highlighted the importance of the trunk in patients with stroke and reported that trunk and barrier dysfunction were associated gait speed. Rehabilitation training of the insula may improve postural control during exercise among patients with stroke [49,50]. The optimal activation of the transverse abdominis increases the intra-abdominal pressure equally in all directions, which activates the entire trunk musculature [51]. Therefore, symmetrical activation of the transverse abdominis and inspiratory muscles is fundamental for dynamic stabilization of the trunk [52]. Courbon et al. [53] suggested that physiotherapy including respiration-related interventions is more effective than general physiotherapy for improving functional activities, such as gait, in patients with stroke.

The results of gait variables in this study revealed no significant differences from the control group; however, significant differences in all items within the study group were observed. Trunk extension posture-based inspiratory muscle training performed in the study group was believed to improve postural stability through symmetrical activation of the transverse abdominis and inspiratory muscles, and based on the improved postural stability, patients exhibited significant results in the gait variables, Timed Up and Go Test, and 10 m walk test.

Because trunk alignment and trunk muscle activity were not directly measured in this study, the underlying biomechanical mechanisms remain speculative. Future studies incorporating objective assessments of trunk alignment and muscle activity are needed to clarify these mechanisms.

## 5. Study Limitations

This study has several limitations that should be acknowledged. First, the sample size was relatively small (*n* = 15 per group), which may limit the generalizability of the findings despite the a priori power calculation. Second, the intervention period was limited to 6 weeks; therefore, the long-term effects of trunk extension-based inspiratory muscle strengthening remain unclear. Third, although efforts were made to minimize bias, complete blinding of participants was not feasible due to the nature of the intervention. Future studies with larger sample sizes and longer follow-up periods are needed to confirm and extend these findings.

## 6. Conclusions

Trunk extension-based inspiratory muscle training may be a promising intervention for improving respiratory function and balance in patients with stroke. The findings suggest that optimizing trunk posture during inspiratory muscle strengthening could enhance thoracic expansion and trunk stabilization. However, further large-scale and long-term studies are required to confirm the clinical effectiveness and generalizability of these results.

## Figures and Tables

**Table 1:** General characteristics of the subjects (*n* = 30).

Variables	SG	CG	*x*/*t*^2^	*p*
	(*n* = 15)	(*n* = 15)		
Gender (M/F, %)	8/7 (53.3/46.7)	8/7 (53.3/46.7)	0.000 ^b^	1.000
Paretic side (R/L, %)	7/8 (46.7/53.3)	7/8 (46.7/53.3)	0.000	1.000
Diagnosis (ICH/IS, %)	9/6 (60/40)	9/6 (60/40)	0.000	1.000
Age (years)	54.33 ± 11.61 ^a^	53.20 ± 15.68	0.225 ^c^	0.824
Height (cm)	164.37 ± 7.40	166.40 ± 6.59	0.756	0.456
Weight (kg)	61.49 ± 9.49	62.87 ± 8.28	0.442	0.676
Onset (months)	15.13 ± 6.68	12.67 ± 6.23	1.046	0.304
BMI (kg/m^2^)	22.66 ± 2.49	22.64 ± 6.23	0.015	0.988
MMSE-K (score)	28.20 ± 1.37	27.93 ± 2.05	0.418	0.679

Note. SG: study group; CG: control group; ICH: intracerebral hemorrhage; IS: ischemic stroke; BMI: body mass index; MMSE-K: Mini-Mental State Examination-Korean version. ^a^ Mean ± standard deviation, ^b^ chi-square test, ^c^ independent *t*-test.

**Table 2:** Homogeneity test of dependent variables (*n* = 30).

Variables	SG	CG	*t*	*p*
	(*n* = 15)	(*n* = 15)		
MIP	41.65 ± 11.57 ^a^	37.24 ± 8.34	1.196 ^b^	0.242
MIFR	2.28 ± 0.74	2.01 ± 0.51	1.154	0.258
MIV	1.04 ± 0.28	1.27 ± 0.44	1.668	0.109
PEF	308.27 ± 100.54	300.89 ± 104.89	0.197	0.846
FEV_1_	1.89 ± 0.56	1.86 ± 0.65	0.118	0.907
BBS	42.67 ± 5.22	41.40 ± 5.32	0.659	0.516
WDR	22.32 ± 11.84	16.50 ± 9.19	1.503	0.144
LOS	33.74 ± 24.75	31.92 ± 26.31	0.195	0.847
TUG	56.62 ± 27.72	57.12 ± 35.49	0.315	0.965
Gait velocity	0.24 ± 0.15	0.27 ± 0.17	0.209	0.836
Cadence	60.01 ± 18.16	63.39 ± 23.88	0.437	0.666
Stride length	45.65 ± 16.49	47.05 ± 15.09	0.242	0.810

Note. SG: study group; CG: control group; MIP: maximum inspiratory pressure; MIFR: maximum inspiratory flow rate; MIV: maximum inspiratory volume; PEF: peak expiratory flow; FEV_1_: forced expiratory volume in 1 s; BBS: Berg Balance Scale; WDR: weight distribution ratio; LOS: limit of stability; TUG: Timed Up and Go Test. ^a^ Mean ± standard deviation, ^b^ independent *t*-test.

**Table 3:** Comparison of respiratory function within and between the groups (*n* = 30).

Variable	Group	Pre	Post	Change (Post-Pre)	^b^ Within-Group *p*	^c^ Between-Group *p*
**MIP (cm H_2_O)**	SG (*n* = 15)	41.65 ± 11.57 ^a^	53.53 ± 9.40	11.88 ± 12.01	0.002	0.002
	CG (*n* = 15)	37.24 ± 8.34	36.67 ± 5.65	−0.58 ± 7.57	0.773	
**MIFR (L/s)**	SG	2.28 ± 0.74	3.07 ± 0.55	0.79 ± 0.75	0.001	0.001
	CG	2.01 ± 0.51	1.97 ± 0.37	−0.04 ± 0.42	0.702	
**MIV (L)**	SG	1.04 ± 0.27	1.31 ± 0.28	0.27 ± 0.40	0.021	0.038
	CG	1.27 ± 0.44	1.22 ± 0.42	−0.04 ± 0.39	0.67	
**PEF (L/min)**	SG	308.27 ± 100.54	355.33 ± 87.58	47.06 ± 82.52	0.044	0.045
	CG	300.89 ± 104.89	299.89 ± 100.88	−1.00 ± 22.59	0.866	
**FEV_1_ (L)**	SG	1.89 ± 0.56	2.34 ± 0.71	0.45 ± 0.67	0.02	0.044
	CG	1.86 ± 0.65	1.87 ± 0.70	0.00 ± 0.49	0.891	

Note. SG: study group; CG: control group; MIP: maximum inspiratory pressure; MIFR: maximum inspiratory flow rate; MIV: maximum inspiratory volume; PEF: peak expiratory flow; FEV_1_: forced expiratory volume in 1 s. ^a^ Mean ± standard deviation; ^b^ Within-group comparison: paired *t*-test; ^c^ Between-group comparison: independent *t*-test based on change scores.

**Table 4:** Comparison of balance within and between the groups (*n* = 30).

Variable	Group	Pre	Post	Change (Post-Pre)	^b^ Within-Group *p*	^c^ Between-Group *p*
**WDR (%)**	SG (*n* = 15)	22.32 ± 11.84 ^a^	11.29 ± 8.38	−11.03 ± 9.56	0.001	0.003
	CG (*n* = 15)	16.51 ± 9.19	14.16 ± 9.01	−2.34 ± 4.02	0.041	
**LOS (cm^2^)**	SG	33.74 ± 24.75	44.14 ± 27.94	10.40 ± 12.75	0.007	0.048
	CG	31.92 ± 26.31	34.19 ± 24.81	2.27 ± 8.27	0.306	
**BBS (score)**	SG	42.67 ± 5.22	46.80 ± 4.57	4.13 ± 2.13	<0.001	0.018
	CG	41.40 ± 5.32	43.27 ± 4.70	1.87 ± 2.77	0.021	

Note. SG: study group; CG: control group; WDR: weight distribution ratio; LOS: limit of stability; BBS: Berg Balance Scale. ^a^ Mean ± standard deviation; ^b^ Within-group comparison: paired *t*-test; ^c^ Between-group comparison: independent *t*-test based on change scores.

**Table 5:** Comparison of gait ability within and between the groups (*n* = 30).

Variable	Group	Pre	Post	Change (Post-Pre)	^b^ Within-Group *p*	^c^ Between-Group *p*
**TUG (s)**	SG (*n* = 15)	56.51 ± 27.72 ^a^	52.75 ± 30.92	−3.87 ± 6.40	0.035	0.972
	CG (*n* = 15)	57.13 ± 35.49	53.35 ± 32.43	−3.78 ± 6.77	0.048	
**Gait velocity (m/s)**	SG	0.24 ± 0.15	0.30 ± 0.20	0.05 ± 0.07	0.011	0.123
	CG	0.27 ± 0.17	0.29 ± 0.19	0.02 ± 0.06	0.274	
**Cadence (steps/min)**	SG	60.01 ± 18.16	63.41 ± 19.74	3.30 ± 5.92	0.043	0.154
	CG	63.39 ± 23.88	63.50 ± 24.34	0.12 ± 6.36	0.944	
**Stride length (cm)**	SG	46.65 ± 16.49	51.63 ± 21.05	5.98 ± 7.00	0.005	0.304
	CG	47.05 ± 15.09	50.38 ± 16.89	3.33 ± 6.84	0.08	

Note. SG: study group; CG: control group; TUG: Timed Up and Go Test. ^a^ Mean ± standard deviation; ^b^ Within-group comparison: paired *t*-test; ^c^ Between-group comparison: independent *t*-test based on change scores.

## Data Availability

The data presented in this study are available on request from the corresponding author. The data are not publicly available due to privacy and ethical restrictions.

## References

[1] Teixeira-Salmela L.F., Parreira V.F., Britto R.R., Brant T.C., Inácio É.P., Alcântara T.O., Carvalho I.F. (2005). Respiratory pressures and thoracoabdominal motion in community-dwelling chronic stroke survivors. Arch. Phys. Med. Rehabil..

[2] Fugl-Meyer A.R., Linderholm H., Wilson A.F. (1983). Restrictive ventilatory dysfunction in stroke: Its relation to locomotor function. Scand. J. Rehabil. Med. Suppl..

[3] Azevedo I.G., Sousa S.L.D.O., Viana E.D.S.R., Dantas D.D.S., Maciel Á.C.C., Da Câmara S.M.A. (2021). Relationship between symptomatic pelvic organ prolapse and respiratory muscle strength in middle-aged and older women in Northeast Brazil: A cross-sectional study. Physiother. Theory Pract..

[4] Dunsky A. (2019). The effect of balance and coordination exercises on quality of life in older adults: A mini-review. Front. Aging Neurosci..

[5] Rodrigues G.D., Gurgel J.L., Galdino I.d.S., da Nóbrega A.C.L., Soares P.P.d.S. (2020). Inspiratory muscle training improves cerebrovascular and postural control responses during orthostatic stress in older women. Eur. J. Appl. Physiol..

[6] Tayashiki K., Takai Y., Maeo S., Kanehisa H. (2016). Intra-abdominal pressure and trunk muscular activities during abdominal bracing and hollowing. Int. J. Sports Med..

[7] Verheyden G., Ruesen C., Gorissen M., Brumby V., Moran R., Burnett M., Ashburn A. (2014). Postural alignment is altered in people with chronic stroke and related to motor and functional performance. J. Neurol. Phys. Ther..

[8] Dickstein R., Sheffi S., Haim Z.B., Shabtai E., Markovici E. (2000). Activation of flexor and extensor trunk muscles in hemiparesis. Am. J. Phys. Med. Rehabil..

[9] Lima Í.N., Fregonezi G.A., Melo R., Cabral E.E., Aliverti A., Campos T.F., Ferreira G.M. (2014). Acute effects of volume-oriented incentive spirometry on chest wall volumes in patients after a stroke. Respir. Care.

[10] Britto R.R., Rezende N.R., Marinho K.C., Torres J.L., Parreira V.F., Teixeira-Salmela L.F. (2011). Inspiratory muscular training in chronic stroke survivors: A randomized controlled trial. Arch. Phys. Med. Rehabil..

[11] Dall’Ago P., Chiappa G.R., Guths H., Stein R., Ribeiro J.P. (2006). Inspiratory muscle training in patients with heart failure and inspiratory muscle weakness: A randomized trial. J. Am. Coll. Cardiol..

[12] Sutbeyaz S.T., Koseoglu F., Inan L., Coskun O. (2010). Respiratory muscle training improves cardiopulmonary function and exercise tolerance in subjects with subacute stroke: A randomized controlled trial. Clin. Rehabil..

[13] Xiao Y., Luo M., Wang J., Luo H. (2012). Inspiratory muscle training for the recovery of function after stroke. Cochrane Database Syst. Rev..

[14] Downey A.E., Chenoweth L.M., Townsend D.K., Ranum J.D., Ferguson C.S., Harms C.A. (2007). Effects of inspiratory muscle training on exercise responses in normoxia and hypoxia. Respir. Physiol. Neurobiol..

[15] Petrovic M., Lahrmann H., Pohl W., Wanke T. (2009). Idiopathic diaphragmatic paralysis—Satisfactory improvement of inspiratory muscle function by inspiratory muscle training. Respir. Physiol. Neurobiol..

[16] Illi S.K., Held U., Frank I., Spengler C.M. (2012). Effect of respiratory muscle training on exercise performance in healthy individuals: A systematic review and meta-analysis. Sports Med..

[17] Griffiths L.A., McConnell A.K. (2007). The influence of inspiratory and expiratory muscle training upon rowing performance. Eur. J. Appl. Physiol..

[18] Nason L.K., Walker C.M., McNeeley M.F., Burivong W., Fligner C.L., Godwin J.D. (2012). Imaging of the diaphragm: Anatomy and function. Radiographics.

[19] Develi E., Subasi F., Aslan G.K., Bingol Z. (2021). The effects of core stabilization training on dynamic balance and pulmonary parameters in patients with asthma. J. Back Musculoskelet. Rehabil..

[20] MacKay-Lyons M.J., Makrides L. (2002). Exercise capacity early after stroke. Arch. Phys. Med. Rehabil..

[21] Koppers R.J., Vos P.J., Boot C.R., Folgering H.T.M. (2006). Exercise performance improves in patients with COPD due to respiratory muscle endurance training. Chest.

[22] Sezer N., Kutay Ordu N., Tomruk Sutheyaz S., Fusun Koseoglu B. (2004). Cardiopulmonary and metabolic responses to maximum exercise and aerobic capacity in hemiplegic patients. Funct. Neurol..

[23] Aydoğan Arslan S., Uğurlu K., Sakizli Erdal E., Keskin E.D., Demirgüç A. (2022). Effects of inspiratory muscle training on respiratory muscle strength, trunk control, balance and functional capacity in stroke patients: A single-blinded randomized controlled study. Top. Stroke Rehabil..

[24] An K.-B., Jeon H.-J., Chang W.-N. (2022). Effects of Inspiratory Muscle Training on Respiration and Balance in Patients with Stroke: A Pilot Randomized Controlled Trial. Exerc. Sci..

[25] Faul F., Erdfelder E., Lang A.-G., Buchner A. (2007). G* Power 3: A flexible statistical power analysis program for the social, behavioral, and biomedical sciences. Behav. Res. Methods.

[26] Weir C.J., Lees K.R. (2003). Comparison of stratification and adaptive methods for treatment allocation in an acute stroke clinical trial. Stat. Med..

[27] Campbell M.K., Elbourne D.R., Altman D.G. (2004). CONSORT statement: Extension to cluster randomised trials. BMJ.

[28] Lee K.-B., Kim M.-K., Jeong J.-R., Lee W.-H. (2016). Reliability of an electronic inspiratory loading device for assessing pulmonary function in post-stroke patients. Med. Sci. Monit. Int. Med. J. Exp. Clin. Res..

[29] Berg K., Wood-Dauphinee S., Williams J. (1995). The Balance Scale: Reliability assessment with elderly residents and patients with an acute stroke. Scand. J. Rehabil. Med..

[30] Morris S., Morris M.E., Iansek R. (2001). Reliability of measurements obtained with the Timed “Up & Go” test in people with Parkinson disease. Phys. Ther..

[31] Dobkin B.H. (2006). Short-distance walking speed and timed walking distance: Redundant measures for clinical trials?. Neurology.

[32] Van Loo M., Moseley A., Bosman J., De Bie R., Hassett L. (2004). Test–re-test reliability of walking speed, step length and step width measurement after traumatic brain injury: A pilot study. Brain Inj..

[33] Kubo H., Nozoe M., Yamamoto M., Kamo A., Noguchi M., Kanai M., Mase K., Shimada S. (2020). Recovery process of respiratory muscle strength in patients following stroke: A Pilot Study. Phys. Ther. Res..

[34] Lanini B., Bianchi R., Romagnoli I., Coli C., Binazzi B., Gigliotti F., Pizzi A., Grippo A., Scano G. (2003). Chest wall kinematics in patients with hemiplegia. Am. J. Respir. Crit. Care Med..

[35] Narain S., Puckree T. (2002). Pulmonary function in hemiplegia. Int. J. Rehabil. Res..

[36] Zhang X., Zheng Y., Dang Y., Wang L., Cheng Y., Zhang X., Mao M., Lu X. (2020). Can inspiratory muscle training benefit patients after stroke? A systematic review and meta-analysis of randomized controlled trials. Clin. Rehabil..

[37] Pozuelo-Carrascosa D.P., Carmona-Torres J.M., Laredo-Aguilera J.A., Latorre-Román P.Á., Párraga-Montilla J.A., Cobo-Cuenca A.I. (2020). Effectiveness of respiratory muscle training for pulmonary function and walking ability in patients with stroke: A systematic review with meta-analysis. Int. J. Environ. Res. Public Health.

[38] Messaggi-Sartor M., Guillen-Solà A., Depolo M., Duarte E., Rodríguez D.A., Barrera M.-C., Barreiro E., Escalada F., Orozco-Levi M., Marco E. (2015). Inspiratory and expiratory muscle training in subacute stroke: A randomized clinical trial. Neurology.

[39] Kim J., Park J.H., Yim J. (2014). Effects of respiratory muscle and endurance training using an individualized training device on pulmonary function and exercise capacity in stroke patients. Med. Sci. Monit. Int. Med. J. Exp. Clin. Res..

[40] Yoo H.-J., Pyun S.-B. (2018). Efficacy of bedside respiratory muscle training in patients with stroke: A randomized controlled trial. Am. J. Phys. Med. Rehabil..

[41] Alfieri F.M., Riberto M., Lopes J.A.F., Filippo T.R., Imamura M., Battistella L.R. (2016). Postural control of healthy elderly individuals compared to elderly individuals with stroke sequelae. Open Neurol. J..

[42] Jung N.-J., Na S.-S., Kim S.-K., Hwangbo G. (2017). The effect of the inspiratory muscle training on functional ability in stroke patients. J. Phys. Ther. Sci..

[43] de Almeida I.C.L., Clementino A.C.C.R., Rocha E.H.T., Brandão D.C., de Andrade A.D. (2011). Effects of hemiplegy on pulmonary function and diaphragmatic dome displacement. Respir. Physiol. Neurobiol..

[44] Hodges P.W., Gandevia S.C. (2000). Changes in intra-abdominal pressure during postural and respiratory activation of the human diaphragm. J. Appl. Physiol..

[45] Saunders S.W., Rath D., Hodges P.W. (2004). Postural and respiratory activation of the trunk muscles changes with mode and speed of locomotion. Gait Posture.

[46] Dorsch S., Ada L., Canning C.G., Al-Zharani M., Dean C. (2012). The strength of the ankle dorsiflexors has a significant contribution to walking speed in people who can walk independently after stroke: An observational study. Arch. Phys. Med. Rehabil..

[47] Ng S.S., Hui-Chan C.W. (2012). Contribution of ankle dorsiflexor strength to walking endurance in people with spastic hemiplegia after stroke. Arch. Phys. Med. Rehabil..

[48] Verheyden G., Vereeck L., Truijen S., Troch M., Herregodts I., Lafosse C., Nieuwboer A., De Weerdt W. (2006). Trunk performance after stroke and the relationship with balance, gait and functional ability. Clin. Rehabil..

[49] Lee K., Park D., Lee G. (2019). Progressive respiratory muscle training for improving trunk stability in chronic stroke survivors: A pilot randomized controlled trial. J. Stroke Cerebrovasc. Dis..

[50] Yoon H.S., Cha Y.J., You J.S.H. (2020). Effects of dynamic core-postural chain stabilization on diaphragm movement, abdominal muscle thickness, and postural control in patients with subacute stroke: A randomized control trial. NeuroRehabilitation.

[51] Frank C., Kobesova A., Kolar P. (2013). Dynamic neuromuscular stabilization & sports rehabilitation. Int. J. Sports Phys. Ther..

[52] Jiroumaru T., Wachi M., Noguchi S., Ikeya M., Hattori T., Fujitani R., Suzuki M., Tanida S., Shichiri N., Fujikawa T. (2021). Is the diaphragm thickness related to gait speed in patients with hemiplegia caused by cerebrovascular accident?. J. Phys. Ther. Sci..

[53] Courbon A., Calmels P., Roche F., Ramas J., Rimaud D., Fayolle-Minon I. (2006). Relationship between maximal exercise capacity and walking capacity in adult hemiplegic stroke patients. Am. J. Phys. Med. Rehabil..

